# Supplementary material for: Electrostrictive microelectromechanical fibres and textiles
Source: Nat Commun. 2017 Nov 10;8:1435. doi: 10.1038/s41467-017-01558-5 (PMC5681540; doi:10.1038/s41467-017-01558-5)
Supplement: Supplementary file 1 — Supplementary Information [file 41467_2017_1558_MOESM1_ESM.pdf]

Supplementary Note

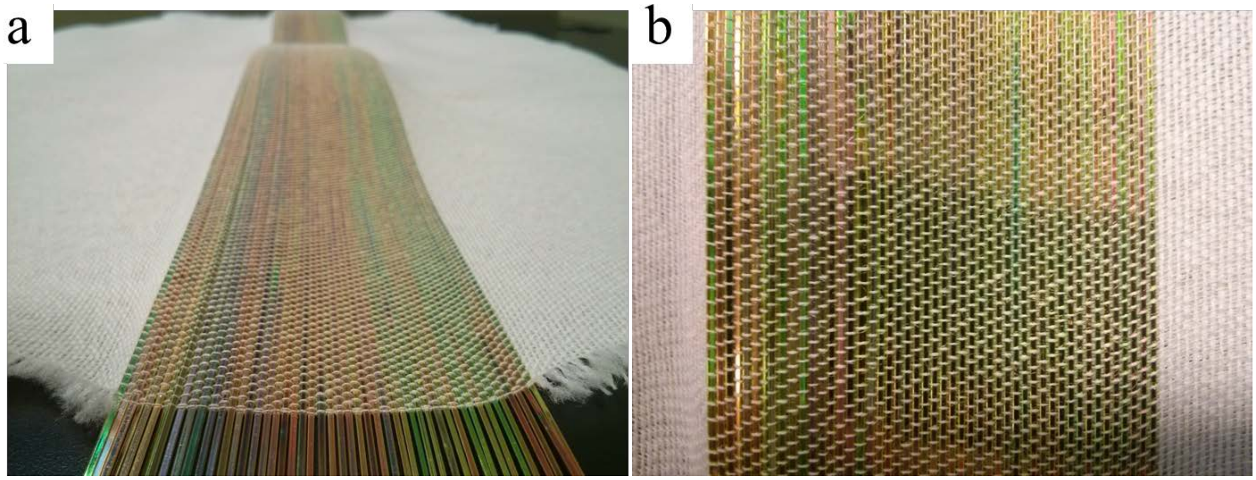

Supplementary Figure 1. MEMS Textile. (a) To date, textiles serve primarily passive functions. Here, for the first time we demonstrate a MEMS textile system, in which the MEMS functionality is embedded directly at the fiber level. This is an enabling capability that could have many interesting applications in advanced functional textiles, including holographic display technologies, on-demand drug release for health and security purposes, etc. The thermal fiber drawing method uniquely satisfies high-throughput, uniformity and flexibility requirements of textile fibers. MEMS behavior is confirmed after the weaving process. (b) A zoomed-in image of textile MEMS.

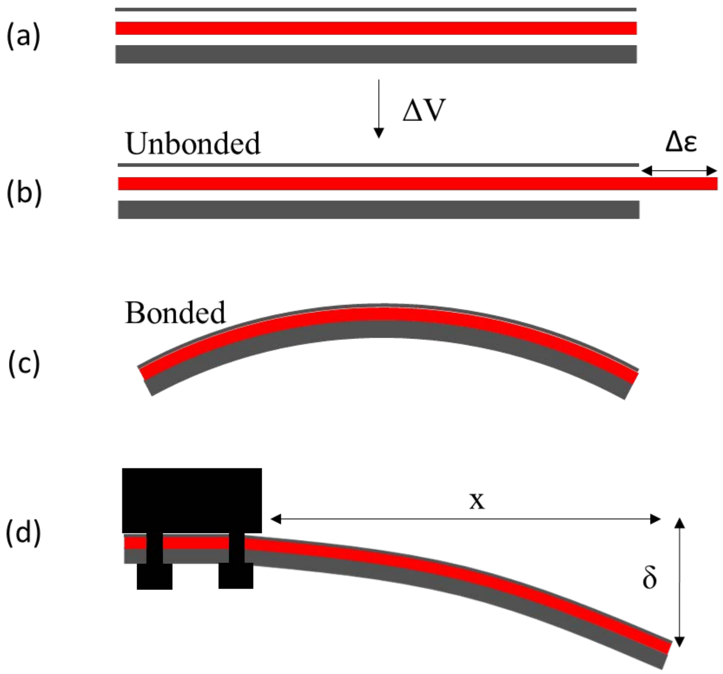

Supplementary Figure 2. Deflection of electrostrictive multimaterial fiber. (a) Two different polymer polycarbonate (passive) and PVDF-TrFE-CFE (active) are shown as grey and red color respectively. (b) Under applied voltage, active layer experiences straining along longitudinal fiber axis. In unbonded case, we can observe clear difference between length of the passive and the active layer after elongation. (c) However in the bonded case this mismatch is compensated by bending of the whole structure towards the direction which is determined by the stength and the dimensions of top and bottom layers. (d) In cantilever beam case (which represents our profilometry setup) bending happens in free edge of the fiber via couple moment.

Curvature κ (inverse of radius of curvature) for cantilever beam case can be measured by the following relationship;

$$\kappa = \frac{2\sin\left[\tan^{-1}\delta/x\right]}{\sqrt{x^2+\delta^2}} \qquad (1)$$

The curvature can also be estimated using material properties and dimensions through the following equation;

$$\kappa = \frac{6t_{\mathrm{pas}}(t_{\mathrm{pas}}+t_{\mathrm{act}})M_{31}V^2}{t_{\mathrm{act}}[\alpha t_{\mathrm{act}}^4+2t_{\mathrm{act}}t_{\mathrm{pas}}(2t_{\mathrm{act}}^2+3t_{\mathrm{act}}t_{\mathrm{pas}}+2t_{\mathrm{pas}}^2)+\frac{t_{\mathrm{pas}}^4}{\alpha}]} \qquad (2)$$

where, $\alpha = \frac{E_{\mathrm{act}}w_{\mathrm{act}}}{E_{\mathrm{pas}}w_{\mathrm{pas}}}$. Subscript *act* and *pas* refer to active and passive layer respectively. *E, w, t*, represents Young`s modulus, width and thickness of material respectively. *V* and M_{31} refer to applied voltage (through thickness of active layer) and electrostriction coefficient for longitudinal fiber axis respectively. Curvature can be found as $\kappa = 0.136\, m^{-1}$ from (1). Then we extract M_{31} from (2) as ~10^{-17} $V^2 \cdot m^{-2}$. During these calculations, we ignore effect of CPE contacts since its Young modulus and dimension are smaller than PC material (passive layer) with order of magnitude. In addition, we ignore top passive layer by subtracting it from bottom passive layer. Namely, thickness of bottom passive layer is around 250 μm and top layer is ~50 μm. Therefore, we assume presence of only bottom layer with 200 μm.

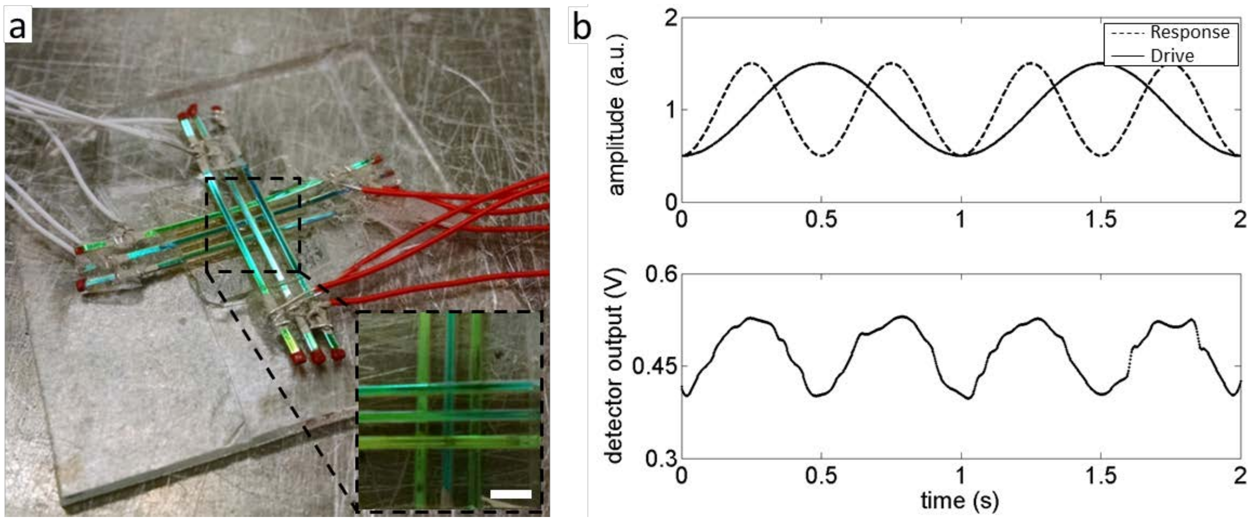

Supplementary Figure 3. Array of fiber MEMS for optical modulation. **(a)** Fiber array embedded in PDMS polymer. Scale bar is 2.5 mm. **(b)** Top: illustration of characteristic frequency-doubling effect from electrostriction effect. A driving frequency of 1 Hz results in a response frequency of 2 Hz. Bottom: modulation of incident optical signal on a fiber array embedded in PDMS. The applied voltage is 300 V at a driving frequency of 1 Hz. Modulation depth is 12.9% for a fiber-to-sensor distance of 40 cm.

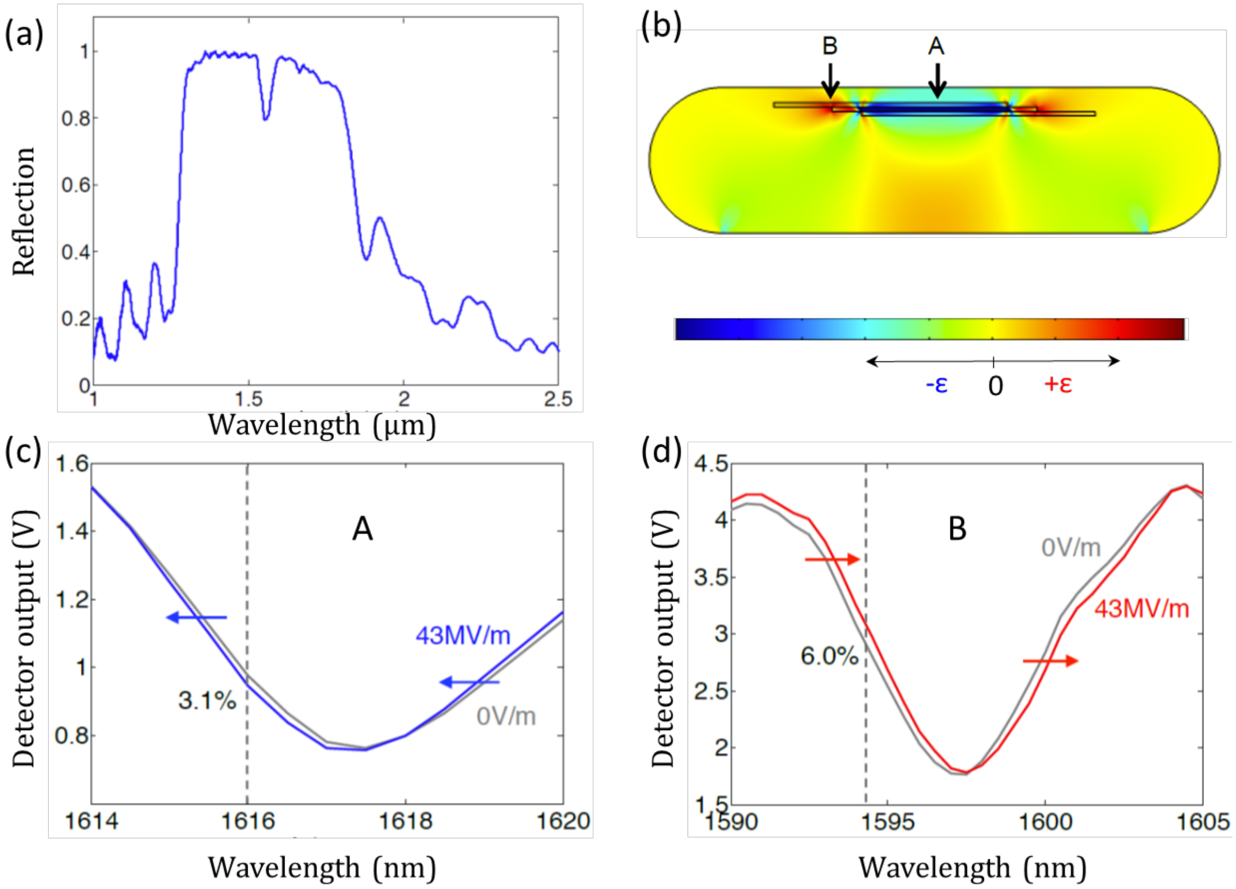

Supplementary Figure 4. (a) FTIR measurement of the reflection spectrum of the Bragg cavity structure with a resonance frequency at 1558 nm. (b) COMSOL simulation of the electric-field induced thickness strain in the hybrid electrostrictive Bragg fiber under an applied voltage of 300 V. (c) Electric-field induced blueshift of the Bragg cavity resonance. (d) Electric-field induced redshift of the Bragg cavity resonance.

A broadband fiber reflection spectrum is measured with a Fourier-transform infrared (FTIR) spectrometer (Bruker Tensor 37 spectrometer with Hyperion FTIR microscope). The result is shown in Supplementary Figure 4a and demonstrates the presence of a photonic bandgap with a resonance around 1550 nm. Simulations of the electric-field induced strain distribution in the fiber structure are performed using the finite-element simulation package COMSOL Multiphysics

(version 4.3). The results of the simulation for the static response show a complex deformation of the fiber structure (Supplementary Figure 4b). The reason for this is that while the Bragg cavity surrounds the entire fiber circumference, the electrostrictive device is restricted to the central portion of the fiber. In the simulations, we assume that the active portion of the electrostrictive layer, *i.e.* the portion of the layer that iscontacted on both sides by a CPE electrode, is ~ 200 μm wide for a total fiber width of ~ 800 μm. This central portion of the fiber (point A in Supplementary Figure 4b) behaves as expected, with a contraction of the electrostrictive layer resulting in a negative strain in the regions above and below it. However, this thickness contraction produces a lateral expansion of the electrostrictive layer due to the Poisson effect, which results in a positive strain in the lateral portions on either side of the terpolymer layer (point B in Supplementary Figure 4b). This leads to both negative and positive strain regions in the Bragg cavity structure along the width of the fiber, with (Supplementary Figure 4b).

To measure these resonance shifts, an optical measurement set-up is built similar to Figure 3c. A tunable laser (ANDO AQ4321D 1520-1620nm) is directed onto the hybrid electrostrictive Bragg fiber, driven by an external high voltage power source. The signal reflected back from the Bragg cavity structure is detected by a photodetector (Thorlabs PDA10CS amplified InGaAs photodetector 700-1800 nm) and recorded with an oscilloscope connected to a computer. A lens with focal length f = 10 cm was used, and the fiber and photodetector were placed at a distance d = 20 cm from the lens, so that the photodetector is in the image plane of the fiber. A 50-μm pinhole is placed in front of the photodetector, restricting the recorded signal to an illuminated area corresponding to a ~ 50 μm diameter spot on the fiber surface. The measurement is performed by sweeping the laser frequency and recording the photodetector output with and without a DC

applied electric field of 43 $MV \cdot m^{-1}$ (which corresponds to an applied voltage of 300 V on a 7 μm-thick electrostrictive layer).

The results are shown in Supplementary Figure 4c,d for two different locations on the fiber surface. In both cases, we observe a measurable effect of the applied electric field on the reflection spectrum in the form of a frequency shift of the resonance. Supplementary Figure 4c shows a shift to shorter wavelengths, while Supplementary Figure 4d features a shift to longer wavelengths, which is consistent with the simulations predicting regions of negative and positive strains in the Bragg structure. This results in a maximum modulation depth of 3.1% at 1616 nm for the blue shift and 6.0% at 1594 nm for the redshift.
