# Supplementary material for: Electrostrictive microelectromechanical fibres and textiles
Source: Nat Commun. 2017 Nov 10;8:1435. doi: 10.1038/s41467-017-01558-5 (PMC5681540; doi:10.1038/s41467-017-01558-5)
Supplement: Supplementary file 3 — Description of Additional Supplementary Files [file 41467_2017_1558_MOESM3_ESM.pdf]

Description of Additional Supplementary Files

File Name: Supplementary Movie 1
Description: **DC deflection case.** When a DC voltage is applied across the CPE electrodes, the P(VDF-TrFE-CFE) layer contracts in the thickness direction and expands in the lateral direction. By deliberately introducing this layer off-centre, asymmetric strain fields can be induced in the fibre, leading to the emergence of transverse-deflection bending modes. The fibre is observed to bend opposite to the face containing the electrostrictive device.

File Name: Supplementary Movie 2
Description: **Resonance at 32 Hz.** By driving the fibre under AC voltage, the electromechanical energy conversion process becomes frequency-dependent and can take advantage of resonance effects. The one edge of the fibre is fixed while another is allowed to vibrate. The amplitude of the oscillations at the resonant frequency point (32 Hz) can be clearly observed.

File Name: Supplementary Movie 3
Description: **Resonance at 18 Hz.** The amplitude of the oscillations at 18 Hz is the highest compared to the other frequency points.

File Name: Supplementary Movie 4
Description: **Amplitude modulation.** It is possible to control the magnitude of oscillations from nm-scale to cm-scale by altering the magnitude of the applied electric field. We demonstrate the tuning of the resonance amplitude for a 7 cm-long fibre by changing the voltage between 0-350 V for the fixed resonance frequency of 20 Hz.
